# Factors influencing mutual support among older people in China: a cross-sectional study

**DOI:** 10.3389/fpubh.2025.1439685

**Published:** 2025-03-05

**Authors:** Lichun Xu, Liyu Lin, Xiaojin Huang, Aixuan Guan, Lianfang Cheng, Yicen Zheng, Shuyi Zhang

**Affiliations:** ^1^Zhongshan Hospital Xiamen University, Xiamen, China; ^2^Xiamen Nursing Association, Xiamen, China; ^3^Xiamen Nursing Quality Control Centre, Xiamen, China; ^4^School of Nursing, Fujian University of Traditional Chinese Medicine, Fuzhou, China; ^5^School of Nursing, Fujian Medical University, Fuzhou, China

**Keywords:** China, older adult, mutual support, eldercare, willingness to participate

## Abstract

**Aims:**

To investigate the willingness of Xiamen’s older adult community members to participate in mutual support for the older adult, and to explore the factors that influence their willingness to participate.

**Background:**

Mutual support in old age care fully respects the autonomy and initiative of the older adult, and has become a practical option for solving the problem of mutual support in old age in China. This study investigates the willingness of Xiamen’s older adult community members to participate in mutual support and scientifically examines the influencing factors behind this willingness.

**Methods:**

This is a cross-sectional study that collected data from September 2023 to January 2024. The study population consisted of 502 older adult people from Xiamen, China, selected using a convenience sampling method. A self-designed questionnaire was used to conduct the survey. Binary logistic stepwise regression analysis was used to determine the influencing factors.

**Results:**

The binary logistic stepwise regression analyses showed that being in a community that regularly organizes activities for the older adult, occasionally participating in community-organized activities for the older adult, being willing to accept help from other older adult people, being willing to provide help to other older adult people, and not living alone were more likely to participate in mutual support.

**Conclusion:**

The government and community organizations should play a role in guiding and safeguarding policies, while also strengthening the promotion of mutual support within the community. Additionally, they should provide targeted and demand-driven mutual support services for the older adult, promoting sustainable and healthy development of mutual support for older people to improve their quality of life and well-being.

## 1 Introduction

The implementation of the family planning policy and the continuous advancement of urbanization have resulted in observable trends in existing family patterns, including an aging population, a decline in the number of children per family, and a reduction in the average size of families. The significant shifts in family structure have contributed to the gradual emergence of empty-nest families as a social norm. Concurrently, the depreciation of currency has resulted in many older adult individuals being unable to rely on savings accumulated during their younger years to support their retirement. Furthermore, the increasing pressure on pension payments faced by the national social security system, coupled with the generally low level of pensions, has further exacerbated the economic pressure on the older adult (1). This phenomenon is not unique to China, as many other countries, especially developed nations, are facing similar challenges with the equally severe issue of population aging.

The physical condition and self-care abilities of the older adult tend to decline with age. Furthermore, the high cost of caregivers and medical care often renders it unaffordable for many older adult individuals. In urban areas, the fast-paced lifestyle, high prices, and intense competition have rendered the traditional family model of older adult care unsustainable. In light of these circumstances, it is evident that the traditional family model of older adult care is no longer capable of fulfilling the diverse needs of the older adult population. Furthermore, the high cost of institutional care and the traditional notion of “raising children to protect the older adult” are challenging for many older adult individuals to accept (2, 3). In response to this challenge, the community-based home care model, which is gradually developing, has been welcomed by the older adult due to its convenience. The community-based home care model represents an older adult care model in which the government assumes control of non-profit organizations, establishes the community as the foundation, the family as the basis, and the institution as the supplement (4). Nevertheless, as a relatively nascent model, it is still confronted with challenges, including inadequate capital investment, an imperfect operational modus operandi, and a dearth of professionalism among service personnel (5). The question of how to achieve the goal of a sense of security, a sense of worthiness, and a sense of joy for the older adult has become a significant societal issue that requires urgent attention.

As the most populous country in the world, China is home to a significant older adult population, and the issue of aging cannot be addressed through policy support alone (6). In light of these considerations, the key to addressing the challenges posed by an aging population in China lies in harnessing the potential of the older adult and exploring innovative models of intergenerational collaboration. The Opinions of the Central Committee of the Communist Party of China and the State Council on Strengthening Efforts to Tackle Population Aging in the New Era indicate that the older adult should be encouraged to continue to play their roles, combining active aging with active aging. Furthermore, the role of the younger older adult should be brought into full play by improving policies and measures on employment, volunteerism, and community governance (7). A novel approach to older adult care, spearheaded by community organizations, is gradually gaining traction. This model upholds the tenet of mutual support and fosters intergenerational care and communication among the older adult.

The Decision of the Central Committee of the Communist Party of China on Further Comprehensive Deepening Reforms and Promoting Chinese Modernisation explicitly proposes to address the issue of an aging population proactively (8). This will be achieved by improving the policy mechanism for the development of the older adult care business and the older adult care industry, and by promoting mutual aid older adult care services. Mutual care can be defined as the mutual support of the older adult by those who are healthy. This can take the form of the older adult helping the older adult who are sick, or it can be a spontaneous initiative whereby groups of older adult people support each other in meeting their own old age needs. This can be seen as belonging to the category of “peer-based older adult mutual support” (9). The mutual care model currently in use in China encompasses several different approaches, including the time bank model, the fixed-point mutual aid model, the twinning mode of help, shared mutual aid, and community volunteer services. Shanghai is the first major Chinese city to experience an aging population, and it is also the first to implement mutual aid programs for the older adult. As early as 1999, the Shanghai community initiated efforts to promote the time bank system. To facilitate the advancement of the mutual care model, the Shanghai Centre for the Development of Aging Care has devised a long-term “old partner” mutual care project, which has been outsourced to social organizations. The concept of mutual care is also being implemented in other cities. For example, Guangzhou has adopted a “time-saving” mutual care model for the older adult, whereby younger, healthy individuals provide care for their older counterparts. Similarly, Wuhan and Qingdao have implemented neighborhood mutual care models. Furthermore, mutual aid is being promoted spontaneously in rural China. Notable examples include the community-based mutual aid for the older adult, which is being promoted by the Fujian Senior Citizens’ Association, and the mutual aid services that have been introduced by the governments of Hebei and Hubei.

In comparison to developing countries, developed countries have experienced an earlier transition to an aging society. The result of years of practice and exploration has been the formation of several mutual support models for the older adult, which are in line with the characteristics of the respective national contexts. For example, the United States has the “village” model (10), Germany has the “multi-generational residence” model (11), Japan has the “neighborhood mutual aid network” model (12), and the “time bank” mutual aid model for the older adult is becoming increasingly popular worldwide (13). The efficacy of the mutual care model for the older adult is evidenced by its capacity to alleviate societal pressure on the older adult, foster community cohesion, and facilitate a shift in attitudes towards the older adult and an enhancement of the older adult service system.

The concept of the “village” in the United States differs from its Chinese counterpart. In the United States, the “village” is a non-profit membership-based volunteer mutual aid organization. The model is primarily comprised of individuals aged 65 and above. In addition to assuming managerial roles, members also serve as volunteers, assisting in meeting fundamental social, physical, emotional, and intellectual needs through mutual aid initiatives. The Village offers a comprehensive range of supportive services and activities, to enhance the quality of life of its members. Additionally, it facilitates access to a diverse array of older adult care services, provided by professional organizations, which enable the older adult to reside in a familiar environment for an extended period. The model encourages contributions from non-profit organizations, with funding derived primarily from membership fees and charitable donations.

The German Federal Government has implemented the multigenerational house model, providing annual funding for its operation and encouraging the participation of charitable organizations and local businesses to ensure multiple sources of funding. The responsibility for the day-to-day management of the “multigenerational house” lies with the community. The three main forms of this model are as follows: firstly, university students share a room with an older person who is unable to afford the high rent, while the older person is offered a spare room free of charge. Secondly, single-parent families share accommodation with the older adult, with the older adult providing childcare and the single-parent families offering companionship and care for the older adult, thus addressing the needs of both parties. Thirdly, the older adult engage in mutual assistance within a familiar environment through the sharing of accommodation. This is done to meet the needs of the older adult. This model serves to dismantle the boundaries between families and generations, thereby establishing a distinctive neighborhood relationship that is worthy of emulation.

One of the most notable examples of a neighborhood support model in Japan is the Suzunokai social organization in Kawasaki City. This is a non-profit organization that has gained considerable recognition for its innovative approach to community development. The membership of the Suzunokai comprises individuals from a variety of age groups and backgrounds, including the older adult, housewives, and other enthusiastic residents of the community. The organization is financed through the contributions of volunteers, social donations, and subsidies from local volunteer centers. The objective is to construct a network of mutual support through mutual assistance among community residents, to establish a relaxed community living environment and harmonious neighborhood relations, and to provide basic life care and nursing services for the older adult and disabled who require home care. The activity space for the neighborhood mutual aid service organized by the “Suzunokai” is not fixed and encompasses a range of locations, including community public activity rooms, residents’ private residences, and some ancillary functions derived from the original function of the commercial space. In Japan, where the phenomenon of empty nesters is relatively prevalent, the Suzunokai is a civil society organization that has attained a high level of specialization and plays a significant role in the operation of the community neighborhood mutual aid model for the older adult.

The development model of “time banks” in the United Kingdom has become more mature. Initially, it provided care services for the older adult and the disabled. Subsequently, it developed into volunteer organizations, which were mainly for retired workers. These organizations met the needs of daily life and spiritual comfort. To facilitate the national development of time banks, the British government has established a comprehensive support policy and financial guarantee mechanism. This encourages the participation of public welfare organizations and charities ensures diversified sources of funding, and promotes the development of social welfare services. Concurrently, the government offers medical training for volunteers and implements personalized service initiatives for diverse demographic groups, which merits emulation. The operational model of the time bank is relatively straightforward. It is managed by community-based administrators for the older adult, and members of the public who are willing to participate can provide services to the older adult and redeem the corresponding service hours through the administration when needed.

The mutual-help older adult care model represents a significant advancement in the field of geriatric care, particularly in light of the demographic shift towards an aging population in China. This assertion is supported by both theoretical inquiry and empirical analysis. The mutual-help older adult care model encompasses a comprehensive range of values, evolving in response to the ongoing development of these models. However, the process of development has also revealed some challenges. For instance, the system is not yet fully developed, and there is still no clear set of standards or norms for the mutual older adult care service model. The activities associated with mutual older adult care have increased the risk for participating older adult individuals, particularly in the context of crisis events, where there is a lack of corresponding legislation and regulations to support them. The current community mutual aid service activities for the older adult commenced with a single source of funding. Financial support from the government for the community to carry out mutual aid activities for the older adult is limited, and the community’s capacity to collaborate and coordinate is also constrained. Consequently, the extant infrastructure for community mutual aid older adult care service activities is inadequate, the content and scope of services are insufficient, and there is a dearth of professional personnel. There is a dearth of government publicity regarding mutual aid for the older adult in urban communities. There is a notable lack of awareness among the older adult population regarding mutual aid initiatives, coupled with a dearth of trust in the capacity of social workers and other traditional-minded older adult individuals to provide adequate support. There is a lack of awareness of the importance of community participation, and the activities provided by the community often fail to meet the real needs of the older adult. This has resulted in a lack of capacity for sustainable development, and the coverage needs to be expanded (14).

The Seventh Population Census of 2020 revealed that the resident population of Xiamen was 5.18 million, with 9.6% of this figure comprising individuals aged 60 and above (15). This proportion is relatively low, reflecting the demographic structure of Xiamen as a special economic zone. However, the situation differs for the household population. In 2020, Xiamen’s household population will be 2.74 million, with 385,400, or 14%, aged 60 or older (16). By 2023, this proportion will have increased to 14.8% (17). This demonstrates that despite the youthful composition of the resident population, the proportion of older individuals in the household registration population is higher and increasing annually. This indicates that Xiamen is confronted with the dual challenge of an aging population and the attraction of a young workforce. In comparison to larger urban centers such as Shanghai (37.4% of the older adult population registered in a household) (18) and Guangzhou (19.38%) (19), Xiamen’s policy on the settlement of the older adult is relatively liberal. It offers a wide range of options for settlement, including educational qualifications, property ownership, and skills. Of particular note is the availability of subsidies for those who settle with a Bachelor’s degree or above. This policy has the effect of promoting a younger demographic profile in the household population. Compared to Quanzhou City, Fujian Province (17.04%) (20) and Zhangzhou City, Fujian Province (19.24%) (21), Xiamen’s status as a special economic zone provides a greater number of employment opportunities, thus contributing to a younger demographic profile. Despite the relatively low proportion of the older adult in Xiamen’s total population, issues such as empty-nester households remain a significant concern. This contrast in the ratio of young to old population serves to highlight the unique social environment faced by the older adult population in Xiamen, situated as it is within the context of rapid urbanization and economic development.

In 2019, Xiamen City selected three communities, namely Yingping Community, Xiange Community in Siming District, and Tieshan Community in Jimei District, as the inaugural cohort of time bank pilot communities to implement the “time bank’s” mutual support for the older adult project. Nevertheless, it remains in the exploratory phase, with relatively slow development, and large-scale promotion has yet to be achieved. Furthermore, a lack of robust management procedures, limited public awareness, and low levels of resident engagement have been identified as significant challenges (22). As the target group for whom the issue of aging is a significant concern, and as the primary source of mutual support within communities, the level of participation among this demographic not only affects their attitudes towards the process of aging but also has a bearing on the future trajectory of mutual support services. Accordingly, this study will investigate and analyze the perceptions, attitudes, and willingness to participate in the mutual care model of older people in the Xiamen community and identify the key factors that promote or impede participation. The objective is to formulate a more targeted policy to enhance older people’s motivation to participate in mutual support and to provide a theoretical basis for the optimization of the community-based older adult care service model in the future. The results of the study will ultimately promote the development of community-based mutual support, enhance the sense of social participation among the older adult, improve their quality of life, and contribute to the construction of a more inclusive and sustainable aging society.

## 2 Relevant concepts and theoretical foundations

### 2.1 Conceptual definition

#### 2.1.1 Community

The term “society” is used to denote a group of people who share close ties and common characteristics. “District” refers to a specific range of activities, while “community” signifies a group of people inhabiting an area with a collectively recognized sense of place, culture, and purpose, and who engage in close interaction. The term “community” denotes a group of people inhabiting a specific geographical area, sharing a common culture and sense of purpose, and engaging in close interaction.

#### 2.1.2 Mutual support among older people

Mutual support for the older adult can be defined as a novel model of old age, characterized by adherence to the principle of voluntary participation, mutual support, and the provision of financial support to the community by the government, the market, social organizations, or the older adult. This model, characterized by mutual assistance between healthier seniors and their less-abled peers, embodies a “peer-cohort support mechanism” within older adult care systems. The utilization of social resources through mutual support for the older adult is a strategy that addresses the deficiencies in the provision of care, offering a comprehensive solution that bridges the gap between institutional and home care.

### 2.2 Theoretical foundation

#### 2.2.1 Theory of active aging

Active aging can be defined as the pursuit of optimal health, participation, and security opportunities for older persons, to improve their quality of life (23). In this context, the term “active” signifies the active involvement of older individuals in social and cultural activities, contingent on their good health. Such participation is identified as a pivotal aspect of active aging, contributing to the maintenance of optimal physical and mental health, and safeguarding human dignity in later life. The concept of active aging encourages the utilization of the ideological heritage, social experience, and professional skills of the older adult, and provides them with the opportunity to participate in society once again, thereby realizing their self-worth and enriching their life in old age. Concurrently, mutual support for the older adult not only alleviates the pressures associated with old age on families and society through mutual support but also enhances their sense of fulfillment and enhances their quality of life. This is consistent with China’s advocacy of active aging, a theory that pervades the entire thesis.

#### 2.2.2 Maslow’s hierarchy of needs theory

Maslow’s hierarchy of needs theory is divided into five levels, which are categorized in the following order: physiological needs, safety needs, social needs, respect needs, and self-actualization needs (24). (1) The first level of this hierarchy encompasses physiological needs, which include food, clothing, housing, transport, and daily care. While the majority of older adult individuals are capable of meeting these needs, those residing in empty nests or with suboptimal self-care abilities may encounter challenges in doing so. (2) Safety needs: with age, physical function declines, and health level varies significantly, so their demand for medical care increases, especially when they are sick and want to get timely treatment. (3) Social needs. The social needs of the older adult are often met by their families, who provide company and emotional support. However, the influence of the general environment has increased the number of empty nesters, with a consequent rise in the number of older adult people living alone, unable to obtain the family warmth and happiness that they desire. In response, these individuals often engage in social activities, seeking both care and a sense of belonging. (4) Respect needs: It is imperative to acknowledge that older individuals aspire to be held in high esteem and acknowledged by their peers. Following their retirement from the primary workforce, older individuals often find themselves transitioning from a position of influence to one marked by vulnerability. This shift in their social role can trigger a psychological response characterized by sensitivity and suspicion, leading them to feel apprehensive about becoming a financial or emotional burden to others. Consequently, older adults often express a strong desire for genuine respect rather than sympathy from their peers. (5) Self-fulfillment needs: Following the satisfaction of this need, they aspire to be a source of assistance to others within a specific domain or facet, through their endeavors. Within the paradigm of mutual assistance for the older adult, this need can be more effectively fulfilled by contributing to the care of other older adult individuals. By leveraging existing resources, mutual support addresses the physical and safety needs of the older adult, while also partially meeting their social interaction, respect, and self-realization needs through intensive recreation, self-management, and mutual support.

#### 2.2.3 Social exchange theory

The social exchange theory, proposed by Homans, is predicated on the integration of psychology and economics, emphasizing that relationships between people are based on interaction and exchange (25). Individuals derive material and spiritual gratification by expending a cost, adhering to the tenets of mutual benefit and reciprocity in the process. Such exchanges are also evident in traditional societies, where values such as “propriety” and “righteousness” are recognized as maintaining social justice. Within the context of mutual support, older adults function as both providers and recipients of care. Through the provision of care and companionship, older people receive care in return. This reciprocal relationship, characterized by the notion of mutual support, is a fundamental aspect of the effective functioning of social networks in old age.

When analyzed through the lens of Maslow’s Hierarchy of Needs Theory and Active Aging Theory, the demand-type factors affecting the willingness of older people to participate in mutual support for the older adult include income, physiological and social needs, and so on. Conversely, the social exchange theory elucidates the supply-type factors that influence older people’s propensity to participate, including community activities, neighborhood mutual support, and economic status. The integration of these three theoretical frameworks provides a comprehensive foundation for examining the participation of older individuals in mutual care within urban communities. Moreover, it offers a robust research basis for investigating the influence of various factors that may shape this participation.

## 3 Materials and methods

### 3.1 Design

This cross-sectional study was conducted in two districts of Xiamen, China, from September 2023 to January 2024.

### 3.2 Participants

This study used convenience sampling in the Siming and Huli districts of Xiamen, China. The inclusion criteria were as follows: age 60 and above, residence in Xiamen for more than 1 year, absence of cognitive, comprehension, or communication disorders, and voluntary informed consent. All participants were fluent in Chinese, understood the questionnaire, and provided written informed consent. A total of 502 older adults from the Xiamen community participated in this study.

### 3.3 Measures

The first draft of the questionnaire was developed based on a literature review (26–28), revised after three expert consultations, and the final version was finalized based on the pilot study. The questionnaire consisted of six sections.

1. Demographic characteristics: gender, age, level of education, marital status, physical condition, self-care ability, and number of chronic diseases.
2. Family structure: number of children, mode of residence, caregivers, harmony in family relationships.
3. Economic status: whether still engaged in income-generating work or activities, source of income, monthly income, whether the main source of income is stable, previous occupation, satisfaction with economic status, whether current income meets old-age needs, whether savings or pension can meet future old-age needs, and whether they want to continue doing simple work if their physical condition allows.
4. Cognitive status: whether they are worried about life, whether they feel lonely, whether they are satisfied with their current mode of aging, their current mode of aging, and what they consider to be the best mode of aging.
5. Social participation and social support: whether there are people who participate in mutual support for the older adult with you, whether there are groups or individuals who engage in activities for the older adult together, whether you have a good relationship with your neighbors, whether the community cares about your life, who you turn to for help when you encounter difficulties, how often the community organizes activities for the older adult, how often you participate in activities for the older adult organized by the community, whether you are satisfied with public facilities in the community.
6. Willingness to participate in mutual support: whether you have learned about mutual support, whether you are willing to receive help from other older people, whether you are willing to give help to other older people, whether you are willing to participate in mutual support, and who you would like to lead the mutual support model.

### 3.4 Data collection

This survey was carried out by four student nurses who had received the same training. They distributed a paper-based questionnaire at locations frequently visited by the older adult in the community, such as parks, community activity centers, supermarket entrances, and community hospitals. The respondents were informed of the study’s purpose and significance before receiving the questionnaires. Respondents gave informed consent before independently completing the questionnaires, with investigators assisting those who had difficulties. A unified guide was used to explain the questionnaires. The completed questionnaires were collected on-site and checked for completeness. Any incomplete questionnaires were filled out immediately.

### 3.5 Data analysis

The collected data were checked for completeness and consistency and then cleaned. Statistical analyses were conducted using SPSS 26.0. Descriptive statistics were used to summarise variables related to general information, family structure, economic status, cognitive situation, social participation, social support, and willingness to participate in mutual support for the older adult. Binary logistic stepwise regression was used to test the predictors of willingness to participate in mutual support in old age. *p* < 0.05 was considered statistically significant.

### 3.6 Ethical considerations

The study was approved by the hospital’s Institutional Review Board, and all procedures followed the 1964 Helsinki Declaration and its subsequent revisions’ ethical standards. Informed consent was obtained from all individual participants included in the study.

## 4 Results

A total of 502 questionnaires were collected and, after excluding invalid questionnaires, there were 491 valid questionnaires, giving an effective response rate of 97.81%.

### 4.1 Demographic characteristics

Out of the 491 older adult cases, 225 (45.8%) were male and 409 (83.3%) were married. The participant’s mean age was 70.51 (SD = 7.022) years. With regards to educational level, 159 (32.4%) had primary school education or below, 139 (28.3%) had junior high school education, 103 (21%) had high school education, and 90 (18.3%) had a bachelor’s degree or higher. In terms of self-assessment of their health, 191 (38.9%) of the older adult rated their health as “very good”, 268 (54.6%) as “fair”, and only 32 (6.5%) as “poor”. 452 (92.1%) of the older adult were fully capable of self-care, while 37 (7.5%) were partially capable. Among the older adult, 176 (35.8%) suffered from one chronic disease, 77 (15.7%) suffered from two chronic diseases, and 39 (7.9%) suffered from three or more chronic diseases simultaneously.

### 4.2 Family status

Regarding the number of children, the majority of older adult people (43.8%) had only one child. 35.2% of older adult people had two or more children, while 18.5% had three or more children. Only 2.4% of older adult people had no children. 12.6% of the older adult population live alone. Out of the total number of older adult individuals, 161 (32.8%) were cared for by their spouses, 19 (3.9%) were cared for by their children, 6 (1.2%) were cared for by babysitters or caregivers, and the remaining individuals took care of themselves. Regarding family harmony, 438 (89.2%) of the older adult had a harmonious relationship with their family members, 48 (9.8%) had an average relationship, and 5 (1.0%) had an inharmonious relationship.

### 4.3 Economic situation

Only 56 individuals (11.4%) remained employed. The majority of older adult individuals (73.5%) relied on pensions as their primary source of income. Additionally, 33 (6.7%) received income from work, 16.1% from child support, and only 3 (0.6%) from social assistance. The proportion of older individuals with a monthly income exceeding $4,000 was highest at 55.8%, and 136 (27.7%) had a monthly income between $2,000 and $4,000. The majority of older people reported having a stable income. Only 43 (8.8%) described their income as average, 8 (1.6%) as relatively unstable, and just 4 (0.8%) as unstable. Of the respondents, 26.5% had professional or government occupations, 23% were company employees, 22.6% were workers, and 15.3% were employed in agriculture. 32.4% of respondents reported being very satisfied with their income, while 41.3% reported being relatively satisfied. 18.9% of the respondents considered their income to be average. Only 5.5 and 1.8% were relatively dissatisfied and very dissatisfied, respectively. In addition, 54.8% of the older adult believed that their current income was sufficient for their needs in old age. Meanwhile, 35.0% believed it barely met their needs, and 4.5% believed it did not meet their needs. Of the older people surveyed, 35.6% believed their savings or pension would meet their future retirement needs, while 45.8% thought it would barely suffice. Furthermore, 22.2% of respondents indicated a preference for continuing to perform simple tasks for as long as their physical condition permits, while 48.5% would not.

### 4.4 Cognitive status

Of the older adult participants, 63.5% reported never feeling worried about their lives, 31.4% reported feeling worried at times, and only 5.1% reported feeling worried a lot. Regarding loneliness, 77.3% reported never feeling lonely, 18.7% reported feeling lonely occasionally, and only 5.3% reported feeling lonely often. Regarding satisfaction with the current mode of aging, 44.6% of respondents reported being more satisfied, 37.7% reported being satisfied, 15.3% considered it to be average, and only 2.2% reported being more dissatisfied. The vast majority of older people age within a family-based context (98.2%). Of those, 81.3% believe that family-based aging is the optimal mode of aging, while only 4.5% believe that mutual support is the superior option.

### 4.5 Status of social participation and social support

Out of the older people surveyed, 11.6% reported having many individuals with whom they could participate in mutual support, while 18.5% reported having one such person. The majority, 69.9%, reported having no one with whom they could participate in mutual support. Additionally, 65.2% of older individuals reported engaging in aging activities with groups or individuals. Regarding neighborhood relations, 49.5% of respondents reported getting along very well with their neighbors, 48.7% reported getting along relatively well, and only 1.8% reported having poor relations with their neighbors. Concerning the well-being of older individuals, 135 (27.5%) respondents reported that the community was often concerned, 242 (49.3%) reported occasional concern, and 114 (23.2%) reported that the community was never concerned with their living situation. In the survey, 32.6% of older adult participants reported that they would first turn to their spouses in case of difficulties. Meanwhile, 51.1% stated that they would first turn to their children or grandchildren, and only a small minority (3.3%) would choose to turn to relatives, friends, or neighbors. The frequency of community-organized activities varied among the surveyed older people, with 17.5% indicating frequent organization, 44% occasional organization, and 38.5% reporting no organization. Regarding participation in community activities, 12.6% of older individuals reported frequent participation, 34.0% reported occasional participation, and 53.4% reported never participating. In terms of satisfaction with public facilities in the community, 66.6% of the older adult expressed relative satisfaction, while 27.5% reported general satisfaction. The publicity for mutual support retirement communities is inadequate. Only 4.7% of older persons reported vigorous publicity in the community, while 77.8% reported no publicity.

### 4.6 Status of participation in mutual support for the older adult

According to the survey, 70.3% of the older adult population reported no awareness of mutual support, 26.7% were somewhat aware and only 3.0% were quite aware. The primary sources of information on mutual support in old age were television, mobile phones, and computers (56.2%). More than half of the older adult population surveyed expressed willingness to accept help from others (53%). The main reasons for their willingness to accept help from older individuals were trust (34.0%), poor health (25.6%), loneliness (15.6%), financial insufficiency (5.7%), and other factors. They were more likely to accept these projects such as emotional comfort such as talking and chatting (51.1%), help with daily living (16.0%), and basic rehabilitation and care (9.5%). However, 47% of older people were unwilling to accept help from others. The main reasons for this were poor health (43.7%), lack of understanding of such services (23.1%), and lack of trust in others (14.8%). Of the older people surveyed, 70.3% stated their willingness to help others in their age group. Specifically, 55.1% were willing to provide emotional support through conversation, while 18.0% were willing to assist with daily activities. The primary motivation for this willingness to help was a desire to support one another, with 80.3% citing this as their reason. However, 29.7% of respondents were not willing to help, with poor health (43.7%) and lack of knowledge about available services (23.1%) being the most common reasons. 76.4% of older individuals expressed willingness to participate in mutual aid for the older adult, while only 23.6% were unwilling. The majority (70.3%) of older individuals prefer the government to lead the mutual aid model for the older adult.

### 4.7 Univariate analysis of factors influencing willingness to participate in mutual support among older adults

**Figure 1:**
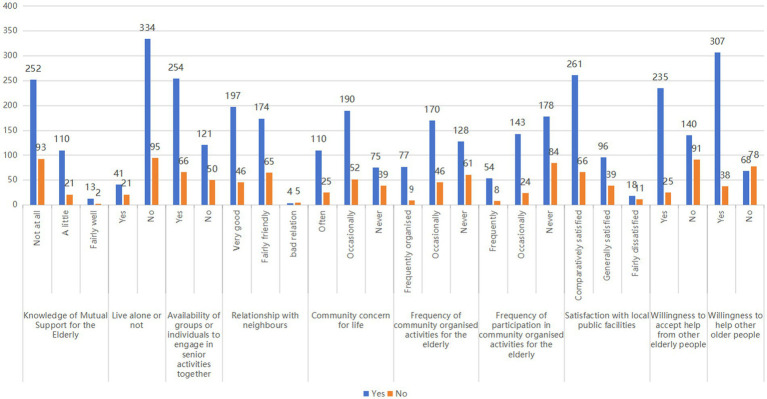
Comparison of willingness to participate in mutual support among older adults across different factors (*n* = 491).

The χ^2^ test results indicate that various factors influence the willingness of the older adult to participate in mutual support in old age, such as whether the individuals are aware of mutual support in old age, whether or not living alone, whether they have a group or individual who does activities together in old age, whether they have a cordial relationship with their neighbors, how well the community cares for their lives, the frequency of community-organized activities for the older adult, how often they participate in community-organized activities for the older adult, how satisfied they are with the community’s public facilities, whether willingness to accept help from other older adult people, and whether willingness to help others, *p* < 0.05 (as shown in Table 1; Figure 1).

**Table 1:** Univariate analysis of factors associated with willingness to participate in mutual support among older adults (*n* = 491).

Variable	Categories	Yes	No	*p*
Knowledge of mutual support for the older adult	Not at all	252	93	0.027
	A little	110	21	
	Fairly well	13	2	
Live alone or not	Yes	41	21	0.042
	No	334	95	
Availability of groups or individuals to engage in senior activities together	Yes	254	66	0.032
	No	121	50	
Relationship with neighbors	Very good	197	46	0.008
	Fairly friendly	174	65	
	bad relation	4	5	
Community concern for life	Often	110	25	0.008
	Occasionally	190	52	
	Never	75	39	
Frequency of community-organized activities for the older adult	Frequently organized	77	9	<0.001
	Occasionally	170	46	
	Never	128	61	
Frequency of participation in community-organized activities for the older adult	Frequently	54	8	<0.001
	Occasionally	143	24	
	Never	178	84	
Satisfaction with local public facilities	Comparatively satisfied	261	66	0.023
	Generally satisfied	96	39	
	Fairly dissatisfied	18	11	
Willingness to accept help from other older adult people	Yes	235	25	<0.001
	No	140	91	
Willingness to help other older people	Yes	307	38	<0.001
	No	68	78	

### 4.8 Binary logistic stepwise regression analysis of willingness to participate in mutual support for the older adult

Independent variables in a binary logistic stepwise regression model contained factors with statistically significant results in the univariate analyses. The study found that older individuals in the community who frequently organize activities for the older adult (OR = 2.688), occasionally participate in community-organized activities for the older adult (OR = 1.939), are willing to accept help from other older individuals (OR = 3.018), are willing to provide help to other older individuals (OR = 5.288), and do not live alone (OR = 2.098) are more likely to participate in mutual aid for the older adult (Table 2).

**Table 2:** Binary logistic stepwise regression analysis of willingness to participate in mutual support for the older adult (*n* = 491).

Variable	B	Std. error	Waldχ^2^	Odd ratio	95% confidence interval
The community regularly organizes activities for the older adult (vs. Never)	0.989	0.402	6.055	2.688	1.223 ~ 5.909
Occasional participation in community-organized activities for older people (vs. Never)	0.662	0.286	5.364	1.939	1.107 ~ 3.395
Willingness to accept help from other older adult people (vs. no)	1.105	0.292	14.294	3.018	1.702 ~ 5.352
Willingness to help other older people (vs. no)	1.665	0.267	38.878	5.288	3.133 ~ 8.926
Not living alone (vs. living alone)	0.741	0.350	4.4890	2.098	1.057 ~ 4.162
Constant	−1.223	0.366	11.156	0.294	

## 5 Discussion

In terms of socio-demographic data, 83.3% of respondents indicated that they were married, a figure that is similar to the results of the survey in other regions of China (29). However, it is generally higher than the results of the community cross-section survey in Italy (49%) (30), Iran (63.2%) (31), Thailand (53.1%) (32), Saudi Arabia (61.3%) (33), and Turkey (68.1%) (34). This discrepancy may be related to the influence of traditional Chinese ideology and cultural values. The literacy level is 67.6%, which is similar to the survey results of 34 cities in 20 provinces in China (66.5% in junior high school and above) (29). It is higher than the survey results of Italy (51.9%) (30) and Turkey (17.8%) (34), and lower than those of Japan (80.4%) (34) and Iran (77.7%) (31). The primary source of income is pension (73.5%), which is higher than the survey results from seven European countries (Germany, Greece, Italy, Lithuania, Portugal, Spain, Sweden) (65.8%) (35). This may be attributed to the fact that the surveyed area is a special economic zone in China and that the majority of older individuals who settled in Xiamen during their early years have had gainful employment and are thus eligible for a pension. However, as these studies only sampled selected cities and populations, their representativeness is limited and the results can only be used as a reference point.

The development of a mutual support model for aging in the community not only alleviates the current situation of inadequate social resources for aging but also provides an important way for groups of older people to realize their self-worth. Mutual support in old age is an important transitional mode in the transformation from the family model of old age to the social model of old age, and can effectively address the realities of the dilemma and provide a useful complementary mode (36).

The study results indicate that only 3.0% of older individuals reported having a good understanding of mutual support in old age, while as many as 70.3% of older individuals in the community had no knowledge at all about mutual support in old age, which is similar to the findings in Guangdong Province (68.69%) (37). The statement implies that the older adult community in Xiamen has not yet embraced mutual support in old age to the desired extent and that the level of awareness among the older adult still needs to be improved. This may be due to the lack of relevant policy support and insufficient social publicity. The survey indicates that the older adult primarily learn about mutual support through media channels such as television, mobile phones, and computers. Therefore, it is recommended to strengthen publicity and education on mutual support for the older adult to enhance their awareness and understanding of this concept. In addition to traditional media like television and mobile phones, it may be beneficial to utilize various channels such as community radio, activity centers for the older adult, health talks, and community volunteers to hold regular lectures and training courses on mutual care for the older adult. These channels can also be used to organize experiential activities on mutual support for the older adult and to encourage the participation of older adult persons in mutual support projects. This will enhance the understanding of the older adult about mutual support and their willingness to participate in such projects. 70.3% of the older adult hope that the government will take the lead in the model of mutual support for the older adult. The intricate and multifaceted nature of the process of fostering mutual support and care for the older adult necessitates the implementation of a comprehensive strategy at the governmental level. If the provision of older adult care services is left to the private sector without the input and support of the government, the range of services available will be quite limited. Thus, the government should play a policy guiding and safeguarding role in this regard, provide legal and institutional safeguards for older adult mutual support, strengthen the construction of older adult care infrastructure, and create a favorable atmosphere for older adult mutual support. Secondly, to promote the sustainable and healthy development of mutual care for the older adult, it is necessary to improve the incentive mechanism. Mutual support cannot rely solely on the unpaid voluntary service model. Voluntary service providers must have a relatively high level of morality and trust. However, in China’s current social context, it is difficult to sustain this model for a long period (38). Therefore, we can learn from the “time bank” model, where service hours can be converted into points that can be exchanged for commodities or corresponding services. This can help the older adult recognize that the costs they have paid can be rewarded accordingly, stimulating their endogenous motivation and enthusiasm to participate in mutual support for the older adult (39). Senior Mutual Support Digital Platform can be utilized by grassroots party organizations as a platform for operational collaboration, facilitating the harmonious integration of party organizations and residents’ community life. This is achieved through a structured approach that encompasses reasonable supervision, planning, and operation, ultimately leading to a mutually beneficial outcome.

76.4% of the older adult are willing to participate in mutual support, which is close to the results of Dong Hui’s survey in Nanchang (69.7%) (40). This study demonstrates the potential for the development of mutual support for the older adult and its promising future. However, it also highlights the urgent need for community workers to find ways to encourage the majority of older adult people in the community to participate in mutual support. The study results indicate that older individuals are more likely to participate in mutual aid for the older adult when their community regularly organizes activities for them, as found in the study by Wang Hao-Lin et al. (41). Furthermore, individuals who participate in community-organized senior activities occasionally are more willing to participate in mutual aid for the older adult than those who never participate, which aligns with Liu Huifeng’s results (42). Frequently organized community activities for the older adult can provide opportunities for communication and social interaction, deepen their knowledge and understanding of each other, and enhance their social ties and sense of belonging (43). Participation in activities can enrich the retirement life of the older adult, exercising their bodies and exchanging thoughts and emotions, which is a manifestation of active aging. Therefore, in the future, efforts should be made to strengthen community building and optimize existing community resources. This includes improving the utilization rate of unused venues and opening them up as places for mutual older adult activities. Simultaneously, it is important to organize diverse activities for the older adult to enrich their daily lives and spiritual well-being. This will fully motivate the older adult in the community to support one another, further enhance their willingness to participate, and create a more harmonious and mutually supportive community atmosphere (27).

Contrary to the results of Hao Ya Ya et al. study (44), older people living alone are less likely to participate in mutual support. This difference may be because Hao Ya Ya investigated rural older adult, while this study focused on older adult individuals in Xiamen. Urban older adult individuals who live alone may be accustomed to living independently and completing daily activities without assistance. They may prefer to remain alone and could be concerned that participating in mutual support may undermine their self-esteem and independence. Additionally, urban elders typically have higher socio-economic status and wider social networks, which may reduce their reliance on others. In the future, older adult care services should be designed to address the characteristics and needs of those living alone. More choices and support should be provided to meet their needs. Additionally, care and concern for older adult people living alone should be strengthened, and a more comprehensive social support system should be established to help them enjoy a better quality of life.

The acceptance of help from others demonstrates an individual’s willingness to receive external support and care when facing difficulties or challenges. It also reflects trust and openness to the goodwill of others. Similarly, the willingness to help others indicates an individual’s social responsibility and generosity, as well as their readiness to provide support and assistance to others. This type of mutual assistance and support not only fosters cohesion and a sense of solidarity within the community but also contributes to the development of more harmonious and stable social relations, promoting the common progress and development of the community (44).

In a survey, 70.3% of older people expressed their willingness to assist other older adult individuals. Their primary motivation was to aid one another, followed by a desire to recognize their self-worth. This percentage is higher than the results of Li Dan’s survey on the willingness of rural older people in Shandong province to help (47.9%) (45). This suggests that the willingness to help each other in old-age care is likely to be higher in an urban setting. The greater willingness of older people in urban areas to assist one another may be attributed to the advantages that urban seniors have in terms of resources, education level, social environment, and economic conditions. Since social resources are more plentiful in cities than in rural areas, older individuals in urban areas are better equipped to assist and are more inclined to do so. Furthermore, older adult individuals residing in urban areas tend to possess higher levels of education and cultural literacy. They demonstrate an increased awareness of and need for mutual support, acknowledging its significance and actively engaging in it. It is noteworthy that the willingness to assist is 5.980 times higher among those who are willing to help compared to those who are not. To further enhance the willingness of the older adult to participate in mutual support programs, the government must formulate a robust policy framework. It is essential that the government ensures the enforcement of organizational regulations and oversees the implementation of mutual assistance activities and treaties while monitoring and evaluating the quality of older adult care services. Concurrently, the enhancement of the content of mutual support services for the older adult and the provision of effective training can bolster the confidence of older individuals in their capacity to provide care for others. The provision of unpaid mutual support is predicated on a high sense of honor, responsibility, and mission. These factors can serve to motivate the older adult to participate in mutual assistance, while also enhancing their sense of belonging and engagement. The introduction of a profit-driven mechanism, such as establishing a “points bank” for mutual support services or transitioning from unpaid mutual support to “low-paid mutual support”, has the potential to motivate a greater number of older individuals to engage in mutual support activities. The promotion of altruism and the reinforcement of the emotional bond between helping others will combine the virtues of altruism and benefit, tradition and modernity, and together they will become the driving force behind mutual and selfless aid services.

53% of the older participants reported their willingness to accept assistance from other older individuals, which is lower than the results found by Wang Jing et al. (74.9%) (46). This difference may be attributed to variations in socio-cultural and community environments between the two studies, as well as the timing of the surveys. The study found that older adult individuals who were willing to accept help were primarily motivated by trust in others and poor health. Conversely, those who were unwilling to accept help cited factors such as poor health, lack of knowledge about the service, and lack of trust in others as their main reasons. The study also revealed that the willingness to participate in mutual aid for the older adult was 2.906 times higher among those who were willing to accept help from others than those who were unwilling to accept help. To enhance the propensity of older individuals to accept assistance, prospective development ought to facilitate a greater prevalence of mutual aid, thereby establishing a dense network of community connections and cultivating a convivial and harmonious community milieu. To this end, it is necessary to establish a community credit system based on the network of neighborhood acquaintances, set up an incentive mechanism for honesty and a disciplinary mechanism for breach of trust, and create a community culture of reciprocal trust (26). Concurrently, the system of record-keeping, application requirements, and time storage should be standardized, and new technologies should be employed to create a “virtual” community acquaintance environment. One potential avenue for achieving this would be the development of an online platform for credit information on mutual assistance for the older adult. Furthermore, communities must conduct regular assessments of the needs of the older adult, gain a comprehensive understanding of their actual requirements, and provide services and support that are tailored to their individual needs (28). Furthermore, awareness-raising and educational campaigns on the acceptance of assistance should be conducted to facilitate an understanding among older individuals that the acceptance of help represents a positive attitude towards life, rather than a state of dependency. This not only enhances the quality of life of older individuals but also fosters the development of deeper social ties.

## 6 Limitations

This study has some limitations. Firstly, the data was collected using convenience sampling, which may result in the sample being under-representative. The use of convenience sampling is contingent upon the accessibility of the researcher, which may introduce sampling bias and limit the extent to which the characteristics of the wider population are reflected. This limitation may impact the external validity of the findings, necessitating caution when interpreting and generalizing the results. Additionally, the definitions of relevant concepts in the questionnaire, such as “mutual support,” are not comprehensive, which may lead to varied interpretations among respondents and impact the clarity of the findings. Future efforts should focus on refining the questionnaire to enhance its ability to yield more objective answers. Moreover, the influence of contextual factors should be emphasized and further investigated, such as whether community size affects older adults’ willingness to participate in mutual support. Furthermore, this study primarily examined the willingness of older adults to engage in mutual support without exploring which specific types of support they prefer. Future research should include a more comprehensive survey involving participants from diverse regions to gain a holistic understanding of their needs and preferences. Finally, future studies could benefit from employing a combination of quantitative and qualitative methods to thoroughly analyze older individuals’ attitudes and needs regarding various forms of mutual support, ultimately promoting the improvement and development of services for the older adult.

## 7 Conclusion

The study results indicate that older individuals who reside in a community that frequently organizes activities for the older adult, occasionally participate in community-organized activities for the older adult, are willing to accept help from other older adult individuals, are willing to provide help to other older adult individuals, and do not live alone are more likely to participate in mutual support. In the future, we should focus on meeting the needs and concerns of the community to formulate more targeted policies and measures. Additionally, we need to increase awareness and promotion of mutual support for the older adult in the community and organize more community activities to guide the awareness of mutual support. In addition, the government and community organizations should play a role in guiding policies and providing safeguards. They should improve the incentive mechanism and establish legal and institutional safeguards for mutual support for the older adult, promote the sustainable and healthy development of mutual support for the older adult, and build a healthier and more active community of mutual support for the older adult to improve the quality of life and sense of well-being of the older adult. The findings of this study are subject to certain constraints, given the variability in policy, environment, and human factors across different locations. Consequently, future initiatives should adopt a targeted approach, aligning strategies with the distinctive characteristics of each region.

## Data Availability

The original contributions presented in the study are included in the article, further inquiries can be directed to the corresponding author.

## References

[1] Bravo JM Ayuso M Holzmann R Palmer E. Addressing the life expectancy gap in pension policy. Insur Math Econ. (2021) 99:200–21. doi: 10.1016/j.insmatheco.2021.03.025, PMID: 39927909

[2] Li F Li L Huang W Zeng Y Long Y Peng J. Assessing the long-term care (LTC) service needs of older adults based on time-driven activity-based costing (TDABC)-a cross-sectional survey in Central China. BMC Nurs. (2024) 23:815. doi: 10.1186/s12912-024-02464-0, PMID: 39516779 PMC11545470

[3] Wang J Wang J Cao Y Jia S Wu B. Older Residents’ perspectives of Long-term care facilities in China. J Gerontol Nurs. (2016) 42:34–43. doi: 10.3928/00989134-20160615-05, PMID: 27319405

[4] Xinwei P Hongchen S. Mutual pension model: its current situation, advantages and development. Theor Explor. (2022) 2:54–60. doi: 10.3969/j.issn.1004-4175.2022.02.008

[5] Wang Y Zhang Q Huang L Zeng F. Factors related to satisfaction with community-based home aging services in Shandong, China. Front Public Health. (2024) 12:1298669. doi: 10.3389/fpubh.2024.1298669, PMID: 38450131 PMC10916510

[6] Luo Y Su B Zheng X. Trends and challenges for population and health during population aging – China, 2015–2050. China CDC Wkly. (2021) 3:593–8. doi: 10.46234/ccdcw2021.158, PMID: 34594944 PMC8393078

[7] Central Committee of the Communist Party of China & State Council. Opinions on strengthening efforts to tackle population aging in the new era [Policy Document]. (2021). Available at: http://www.gov.cn/zhengce/2021-11/24/content_5653181.htm (Accessed 17 February, 2025).

[8] Central Committee of the Communist Party of China. Resolution of the central committee of the communist party of china on further deepening reform comprehensively to advance chinese modernization, (2024). Available at: https://www.gov.cn/zhengce/202407/content_6963772.htm

[9] Wei C Canxian Z Xia NX. Challenges on Chinese traditional pension security mode and explorations on the diversified ways. Theory Mod. (2012) 3:41–6. doi: 10.3969/j.issn.1003-1502.2012.03.006

[10] Graham CL Scharlach AE Stark B. Impact of the village model: results of a National Survey. J Gerontol Soc Work. (2017) 60:335–54. doi: 10.1080/01634372.2017.1330299, PMID: 28509628

[11] Rudel M Abraham M Görtler E. Care preferences and spatial mobility: factors influencing care-related willingness to move of elderly people in partnerships in a rural area. Z Gerontol Geriatr. (2017) 50:200–9. doi: 10.1007/s00391-015-0991-z, PMID: 26650034

[12] Suzuki K Dollery BE Kortt MA. Addressing loneliness and social isolation amongst elderly people through local co-production in Japan. Soc Policy Adm. (2021) 55:674–86. doi: 10.1111/spol.12650

[13] Lee C Burgess G Kuhn I Cowan A Lafortune L. Community exchange and time currencies: a systematic and in-depth thematic review of impact on public health outcomes. Public Health. (2020) 180:117–28. doi: 10.1016/j.puhe.2019.11.011, PMID: 31887608 PMC7093815

[14] Sijia H Bin L. The historical evolution and promotion path of community mutual assistance in the Perspective of urban transformation. Soc Sci Hunan. (2022) 4:120–6.

[15] (NBS) NBoS. Seventh National Population Census Main Data. (2021). Available at: https://www.stats.gov.cn/sj/pcsj/rkpc/d7c/

[16] Network S. Xiamen’s current aging level is 14%, with a total of 15,495 beds. (2021). Available at: https://xm.fjsen.com/2021-02/26/content_30653057.htm

[17] Xiamen PGO. Xiamen municipal people’s congress delegates inspect the construction of the home-based elderly care system. (2023). Available at: https://www.xm.gov.cn/jdhy/wjjd/mtjd/202304/t20230410_2752294.htm

[18] Shanghai Municipal Bureau of Civil Affairs. Release of comprehensive statistical information on the elderly population, aging-related initiatives, and elderly care services in shanghai (2023) (2024). Available at: https://mzj.sh.gov.cn/2024bsmz/20240706/73924c349f4d475a9d46b6019f1a396b.html (Accessed 30 July, 2024).

[19] Government GMP. Guangzhou releases 2023 data on elderly population and aging-related initiatives. (2024). Available at: https://www.gz.gov.cn/zwgk/zdly/mzxx/yljgjbxx/content/post_9900768.html

[20] Bureau QCA. Reply to recommendation no. 1020 of the third session of the 17th municipal People’s congress. (2024). Available at: https://mzj.quanzhou.gov.cn/zwgk/zfxxgkzl/fdzdgknr/yzdgkdqt/202406/P020240605574390000035.pdf

[21] Government FPP. Solving the old-age dilemma and realising ‘a sense of security for the elderly. (2023). Available at: https://www.fujian.gov.cn/xwdt/mszx/202307/t20230706_6200586.htm

[22] Wenjing K Yanzhu G. Explore the “time bank” mutual support elderly model to promote the construction of “love Xiamen”. Xiamen Sci Technol. (2020) 4:5–9.

[23] Dogra S Dunstan DW Sugiyama T Stathi A Gardiner PA Owen N. Active aging and public health: evidence, implications, and opportunities. Annu Rev Public Health. (2022) 43:439–59. doi: 10.1146/annurev-publhealth-052620-091107, PMID: 34910580

[24] Zhang T Liu Y Wang Y Li C Yang X Tian L. Quality indicators for the care of older adults with disabilities in long-term care facilities based on Maslow’s hierarchy of needs. Int J Nurs Sci. (2022) 9:453–9. doi: 10.1016/j.ijnss.2022.09.012, PMID: 36285078 PMC9587388

[25] Ahmad R Nawaz MR Ishaq MI Khan MM Ashraf HA. Social exchange theory: systematic review and future directions. Front Psychol. (2022) 13:1015921. doi: 10.3389/fpsyg.2022.1015921, PMID: 36710813 PMC9878386

[26] Liu B Sun Y. The influence of interpersonal trust on rural Residents’ willingness to participate in mutual aid for the aged: an empirical analysis based on the survey data of Hubei and Henan provinces. Comput Intell Neurosci. (2022) 2022:2366425. doi: 10.1155/2022/2366425 36035844 PMC9410935

[27] Marsh C Agius PA Jayakody G Shajehan R Abeywickrema C Durrant K. Factors associated with social participation amongst elders in rural Sri Lanka: a cross-sectional mixed methods analysis. BMC Public Health. (2018) 18:636. doi: 10.1186/s12889-018-5482-x 29769054 PMC5956789

[28] Tang X Li L Yao K Luo Q Zhao L Li L. Association between social support and mutual-support needs among the rural adults in China: a cross-sectional study. Front Public Health. (2023) 11:1171046. doi: 10.3389/fpubh.2023.1171046 37333532 PMC10275609

[29] Zhang H Loi SM Zhou S Zhao M Lv X Wang J. Dementia literacy among community-dwelling older adults in urban China: a cross-sectional study. Front Public Health. (2017) 5:124. doi: 10.3389/fpubh.2017.00124, PMID: 28638820 PMC5461251

[30] Mulasso A Argiolu L Roppolo M Azucar D Rabaglietti E. Emotion experience and frailty in a sample of Italian community-dwelling older adults. Clin Interv Aging. (2017) 12:2017–24. doi: 10.2147/CIA.S147121, PMID: 29238176 PMC5716397

[31] Nazari S Bakhtiyary M Shabestari AN Sharifi F Afshar PF. Relationship between lifestyle and frailty among Iranian community-dwelling older adults: pilot study. JAR Life. (2023) 12:93–9. doi: 10.14283/jarlife.2023.16, PMID: 38046197 PMC10690137

[32] Thinuan P Siviroj P Lerttrakarnnon P Lorga T. Prevalence and potential predictors of frailty among community-dwelling older persons in northern Thailand: a cross-sectional study. Int J Environ Res Public Health. (2020) 17:4077. doi: 10.3390/ijerph17114077, PMID: 32521642 PMC7312471

[33] Alqahtani BA Alenazi AM Alshehri MM Osailan AM Alsubaie SF Alqahtani MA. Prevalence of frailty and associated factors among Saudi community-dwelling older adults: a cross-sectional study. BMC Geriatr. (2021) 21:185. doi: 10.1186/s12877-021-02142-9, PMID: 33731034 PMC7972196

[34] Korkmaz Aslan G İncİ FH Kartal A. The prevalence of insomnia and its risk factors among older adults in a city in Turkey’s Aegean region. Psychogeriatrics. (2020) 20:111–7. doi: 10.1111/psyg.12464, PMID: 31137084

[35] Eslami B Di Rosa M Barros H Torres-Gonzalez F Stankunas M Ioannidi-Kapolou E. Lifetime abuse and somatic symptoms among older women and men in Europe. PLoS One. (2019) 14:e0220741. doi: 10.1371/journal.pone.0220741, PMID: 31393925 PMC6687146

[36] Zhengjun Z Ling W Yanbo D. Research on innovative design of community mutual aid elderly care service platform based on Kano model. Heliyon. (2023) 9:e15546. doi: 10.1016/j.heliyon.2023.e15546, PMID: 37131443 PMC10149218

[37] Yu Y Yuan Z. Study on the urban elderly people’s willingness to participate in mutual-aid elderly care and influencing factors in Guangdong Province. Soft Sci Health. (2024) 38:35–9. doi: 10.3969/j.issn.1003-2800.2024.03.008

[38] Xuefeng H. Mutual aid for the aged: the way out for the aged in China’s rural areas. J Nanjing Agric Univ. (2020) 20:1–8. doi: 10.19714/j.cnki.1671-7465.2020.0070

[39] Lasker J. Time banking and health: the role of a community currency Organization in Enhancing Well-Being. Health Promot Pract. (2011) 12:102–15. doi: 10.1177/1524839909353022, PMID: 20685912

[40] Hui D. The analysis about the willingness of the old to participate in mutual support: Based on the survey of Nanchang (Master’s thesis). Nanchang, Jiangxi, China: Jiangxi University of Finance and Economics (2018).

[41] Haolin W Qian Y. Analysis on the willingness of community mutual support for the aged and its Influencing Factors in Bengbu City. J Suihua Univ. (2019) 39:21–5. doi: 10.3969/j.issn.2095-0438.2019.11.006

[42] Huifeng L. Study on the willingness and influencing factors of “time bank” mutual pension participation of young elders (Master’s thesis). Guangzhou, Guangdong, China: Guangdong University of Technology (2023).

[43] Nemoto Y Nonaka K Hasebe M Koike T Minami U Murayama Y. Factors that promote new or continuous participation in social group activity among Japanese community-dwelling older adults: a 2-year longitudinal study. Geriatr Gerontol Int. (2018) 18:1259–66. doi: 10.1111/ggi.13457 29998492

[44] Yaya H Hongxia B. Analysis on the willingness and influencing factors of the community mutual support for the Elderly in rural areas in Shandong Province. Northwest Popul J. (2018) 39:96–104. doi: 10.15884/j.cnki.issn.1007-0672.2018.02.013

[45] Dan L. Study on the service willingness of the elderly in mutual Support and its promotion strategies——Example of Shandong Province (Master’s thesis). Tai’an, Shandong, China: Shandong Agricultural University (2022).

[46] Jing W. Study on the influencing factors of the participation Willingness of the elderly in mutual assistance for the elderly in urban communities (Master’s thesis). Kaifeng, Henan, China: Henan University (2023).

